# Supplementary figures and images for: Single-cell analysis of menstrual endometrial tissues defines phenotypes associated with endometriosis
Source: BMC Med. 2022 Sep 15;20:315. doi: 10.1186/s12916-022-02500-3 (PMC9476391; doi:10.1186/s12916-022-02500-3)

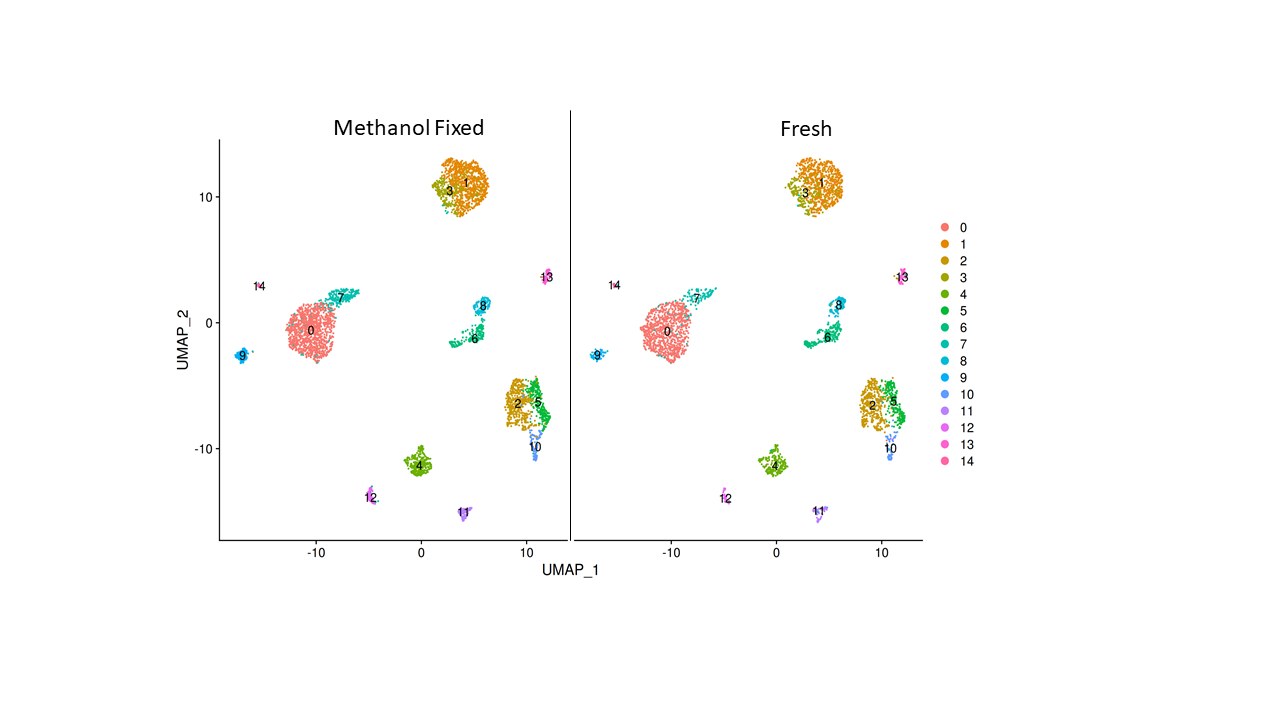

Supplement: Supplementary file 1 — Additional file 1. Comparison of UMAP plots of ME digests using fresh cells vs. methanol fixation. Virtually identical UMAP plots are observed using either fresh or methanol fixed cells from a single subject. The ME samples were prepared by tissue enrichment and tissue digestion, followed by scRNA-Seq analysis, as described in the methods section. [file 12916_2022_2500_MOESM1_ESM.tif]

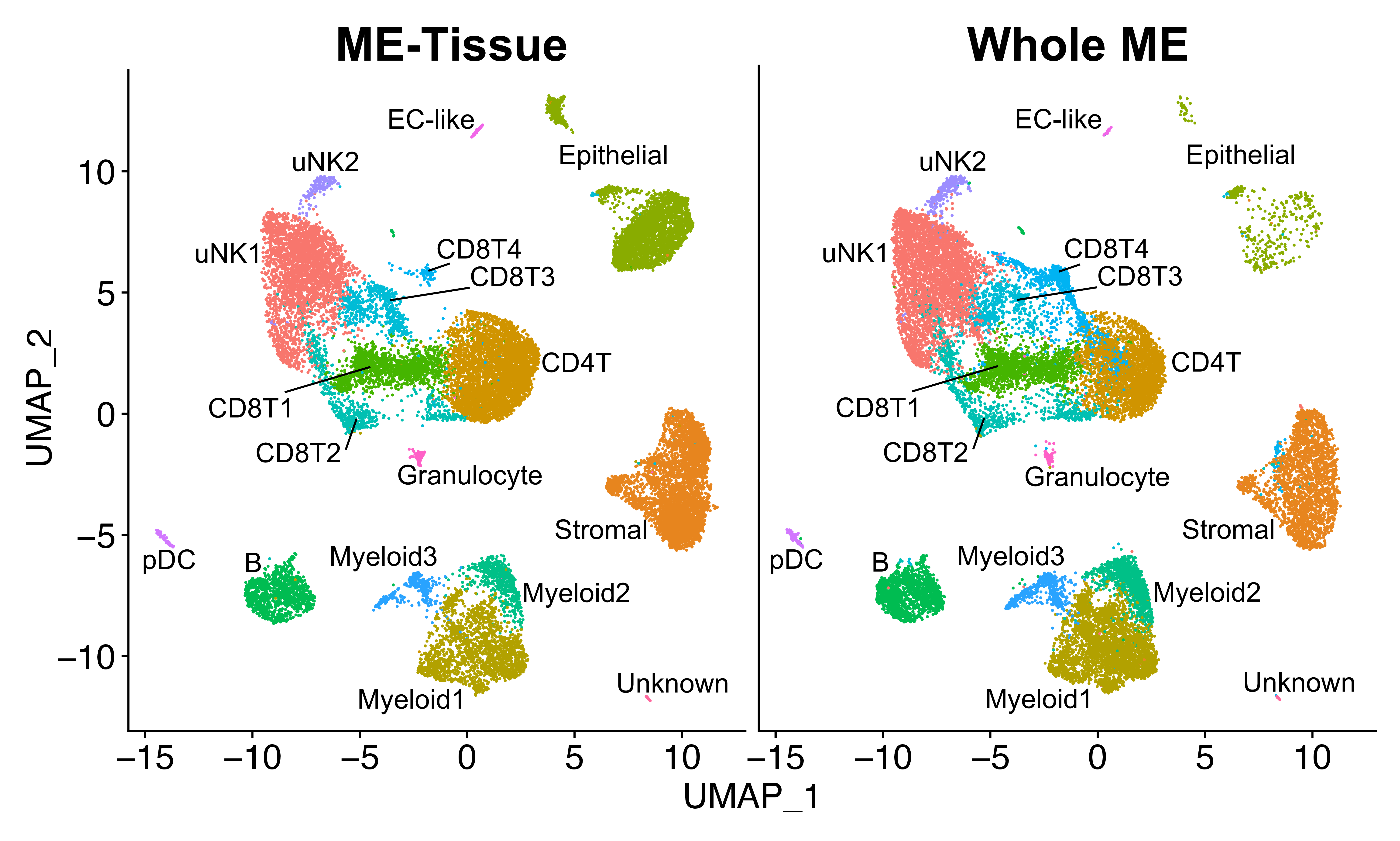

Supplement: Supplementary file 3 — Additional file 3. Cell cluster composition of ME is similar when ME is analyzed after enrichment for endometrial tissues and digested or analyzed as digested whole ME. Comparison of UMAP plots is shown for ME samples prepared by tissue enrichment of menstrual effluent (“ME-Tissue”; 6 diagnosed subjects and 5 controls) or when tissue digestion is applied to unfractionated ME (“whole ME”, 5 diagnosed subjects, and 4 controls). The various cell types are generally well represented between the two approaches to ME preparation. Of note there is an increased yield of epithelial cells in ME samples enriched for tissue. The positive gene markers used to generate the cell clusters shown are included in Additional file 2. [file 12916_2022_2500_MOESM3_ESM.tiff]

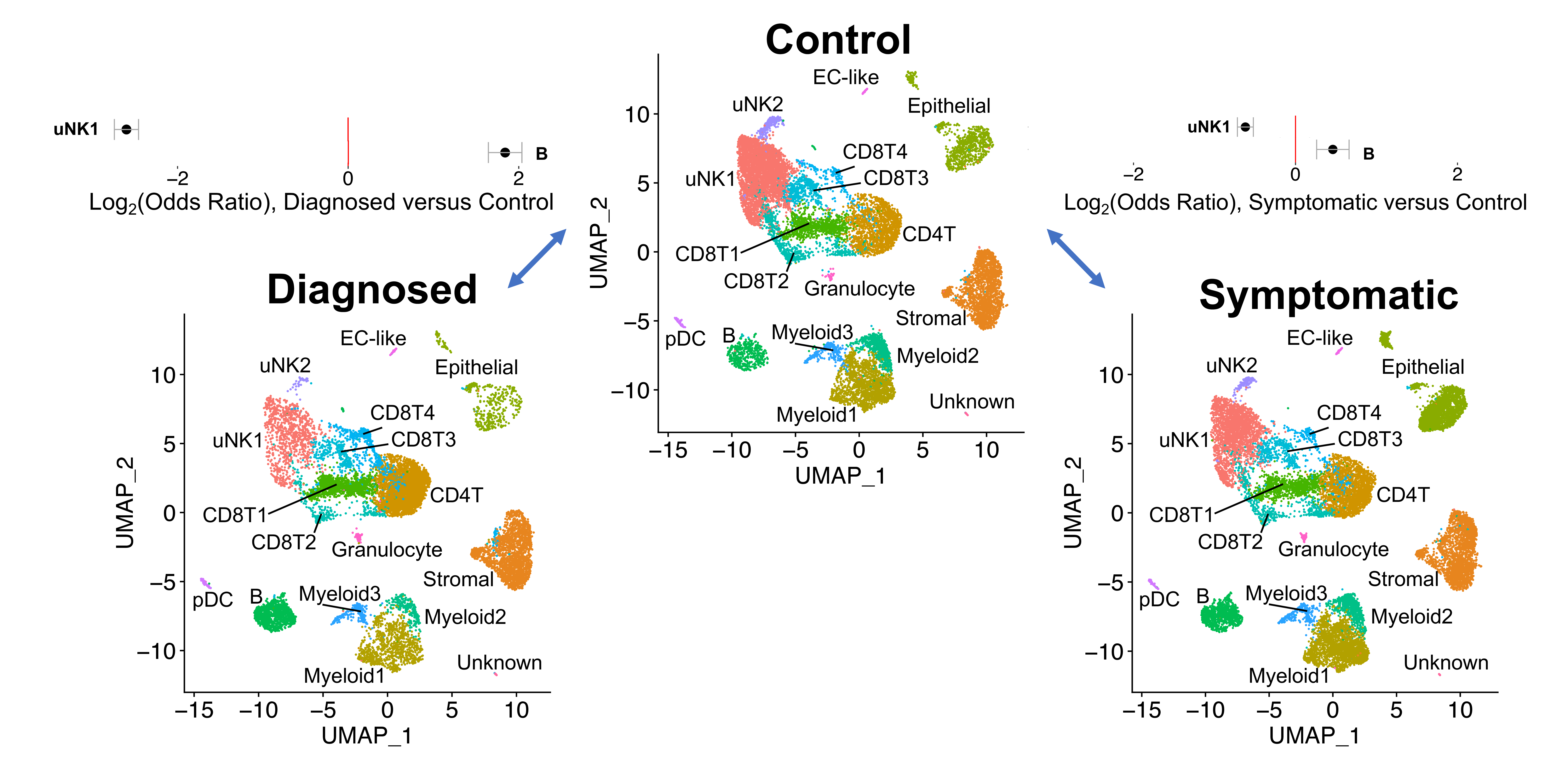

Supplement: Supplementary file 4 — Additional file 4. Cell clusters of ME samples distinguish endometriosis cases and symptomatic cases vs. controls. The combined UMAP plot shown in Fig. 1 is split into controls (n = 9), cases (n = 11), and subjects with suggestive symptoms of endometriosis but without laparoscopic tissue diagnosis – the “symptomatic” group (n = 13). Comparisons of uterine NK (uNK) cell and B cell frequencies in the symptomatic group show a trend that is similar to cases vs. controls. The positive gene markers used to generate the cell clusters shown are included in Additional file 2. [file 12916_2022_2500_MOESM4_ESM.tiff]

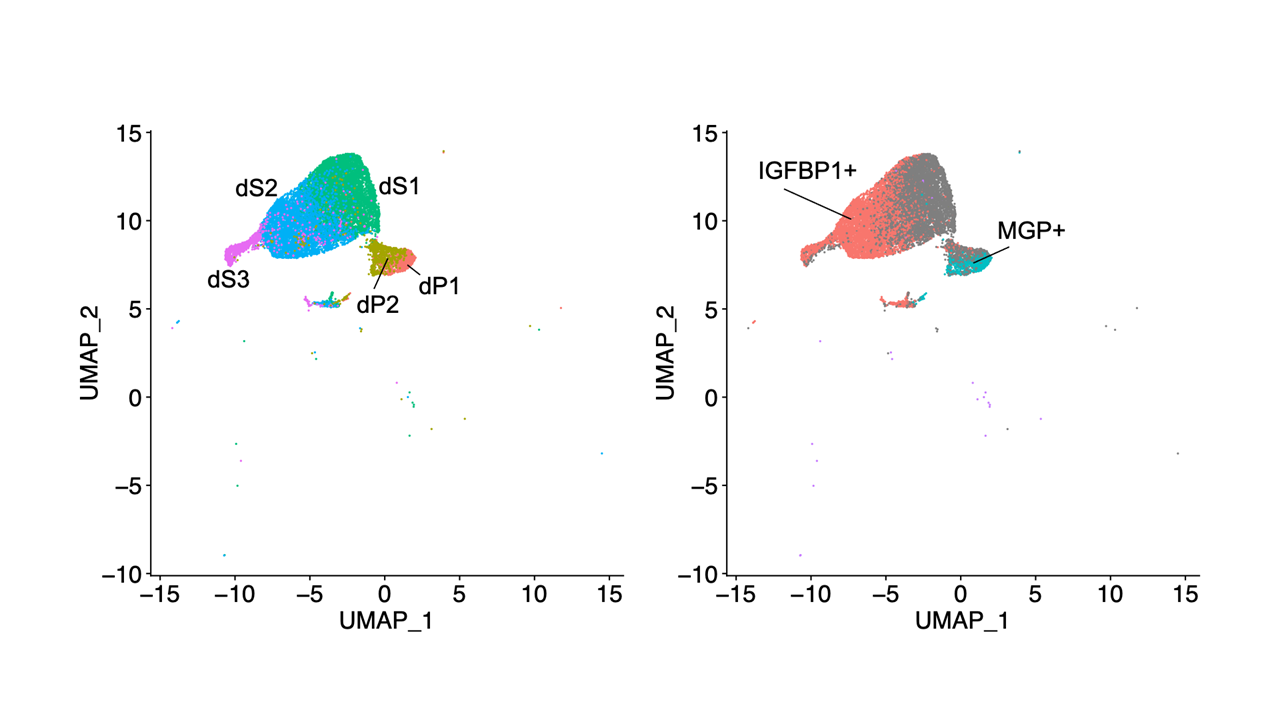

Supplement: Supplementary file 7 — Additional file 7. Stromal cell subclusters in ME samples map to stromal cell clusters found in first trimester decidua. We have compared the mapping of stromal subclusters reported by Vento-Tormo [26], based on the analysis of decidua in the first trimester, with the mapping of stromal cell subclusters we have described in menstrual effluent (ME). Note that our IGFBP1+ subcluster maps almost identically to the decidualizing stromal cell subset dS2 defined by Vento-Tormo [26]. In addition, our MGP+ subset shows a substantial overlap with dP2 and dP1 of Vento Tormo [26], subsets which are attributed to the perivascular stromal cells in first trimester decidua. [file 12916_2022_2500_MOESM7_ESM.tif]

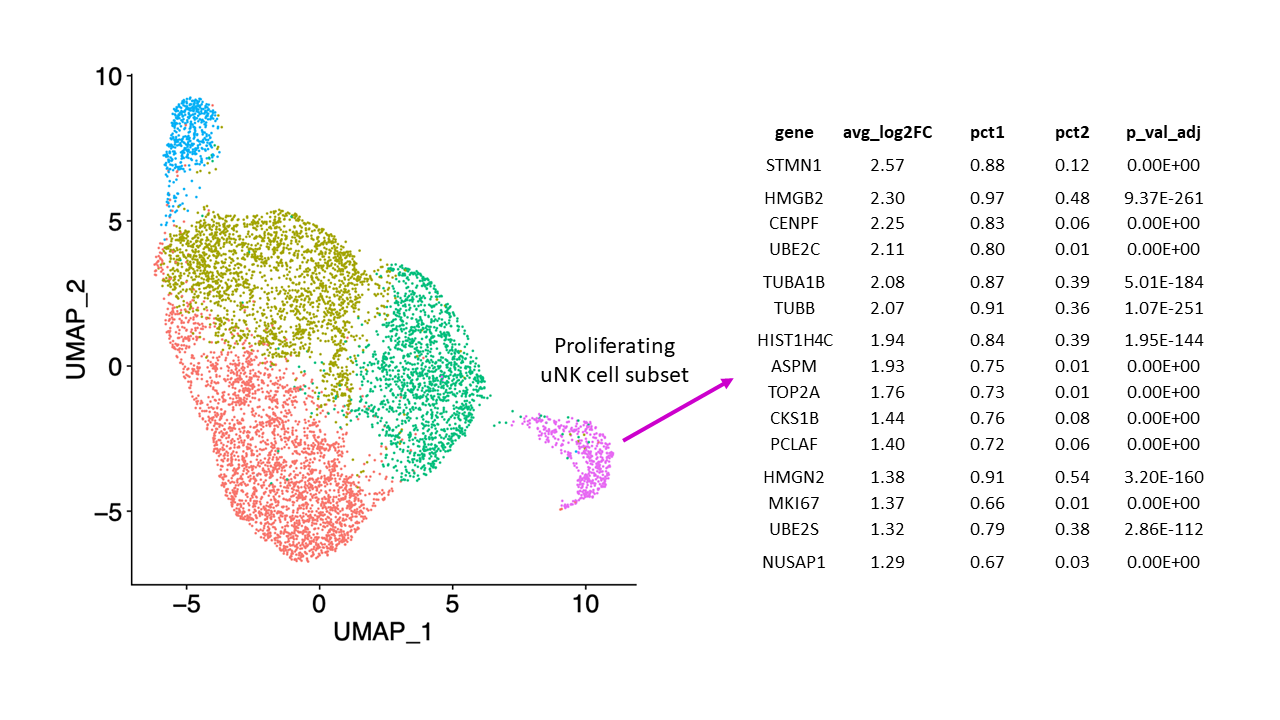

Supplement: Supplementary file 8 — Additional file 8. uNK subclusters reveal a proliferating uNK subcluster enriched in control ME. We have examined subclusters of uterine NK (uNK) cells in our dataset and identified a subcluster whose gene expression patterns reflect cell proliferation, with substantial enrichment of MKI67 and TOP2A. This subset is over 98% matched to a proliferative uNK cell subcluster defined in the decidua of first trimester pregnancy [26]. This subset corresponds to our subset uNK2 that is enriched in controls (Fig. 3). [file 12916_2022_2500_MOESM8_ESM.tif]

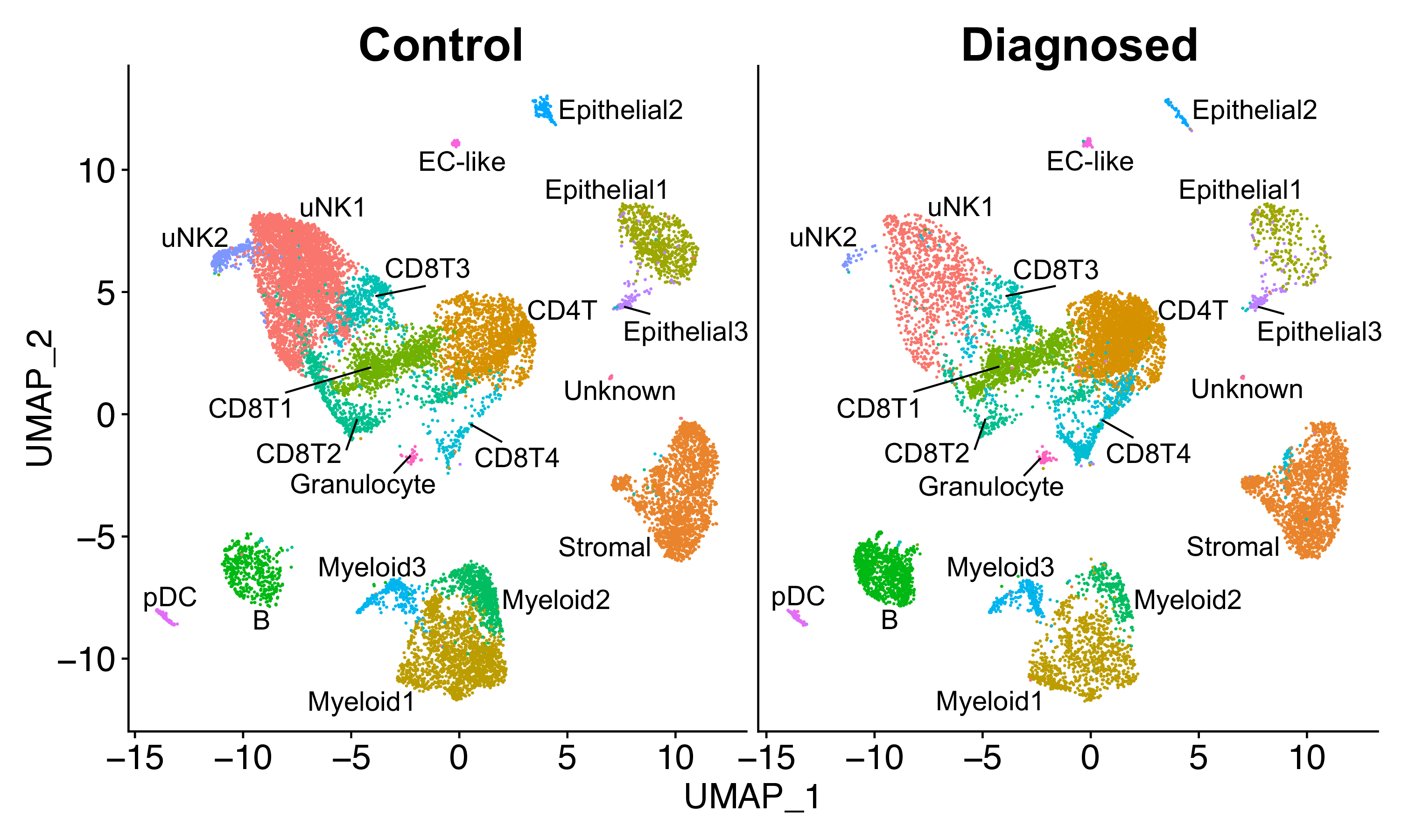

Supplement: Supplementary file 9 — Additional file 9. The UMAP plot derived from a reanalysis of endometriosis cases (n=10) and controls (n=9) after removal of one subject on hormones. For this reanalysis, a total of 1112 singlet cells were eliminated from the ME-tissue run from one affected subject on hormones. Only the singlets were analyzed for this revised figure which shows only subtle changes in the details of the UMAP shown in Fig. 3 of the main text. [file 12916_2022_2500_MOESM9_ESM.tiff]

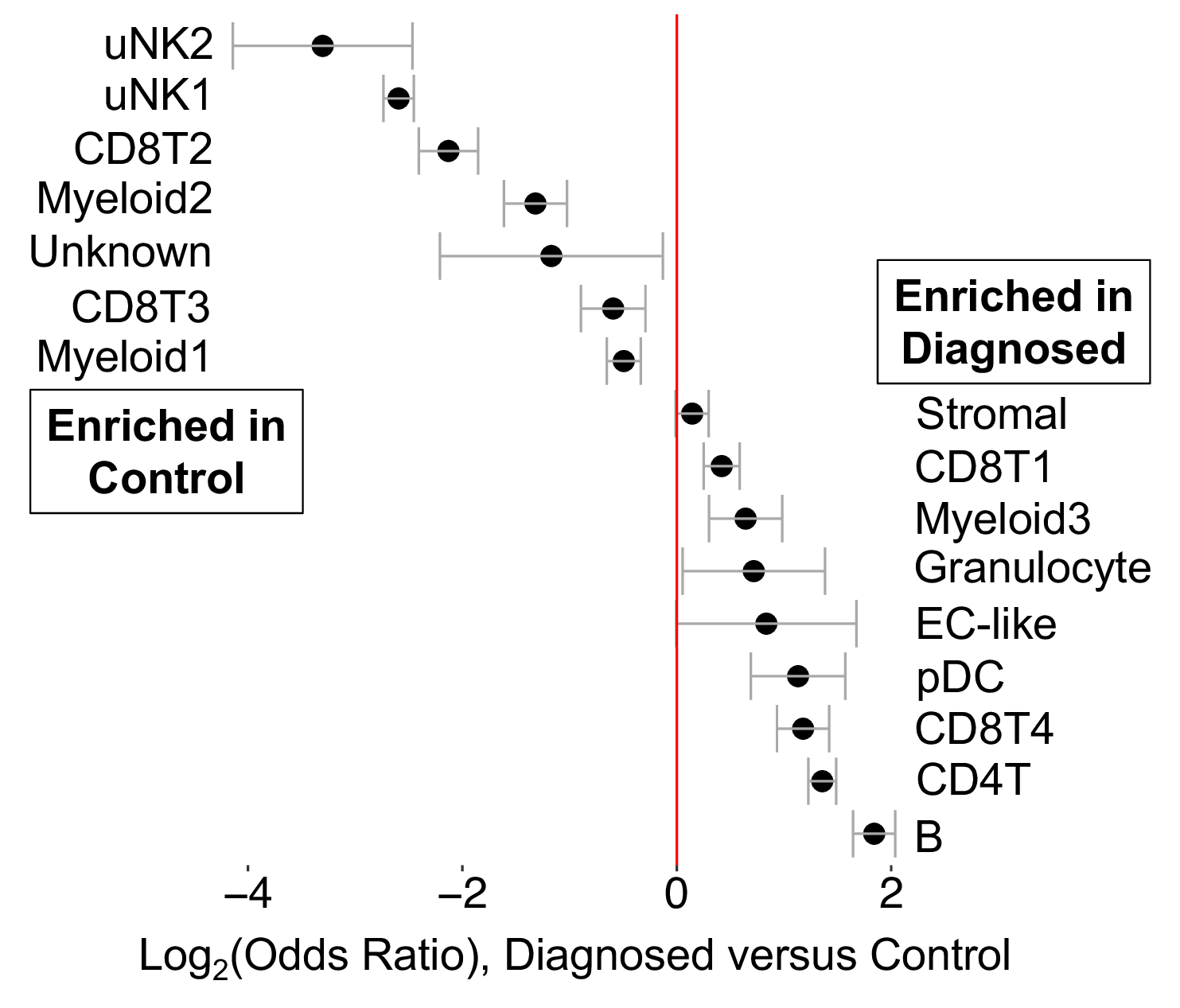

Supplement: Supplementary file 10 — Additional file 10. A reanalysis of endometriosis cases and controls after removal of one subject on hormones. The Log2 odd ratios (OR) with cell subsets enriched in controls (n=9) on the left and cell subsets enriched in cases (n=10) on the right. As is the case for Fig. 4 in the main text, it is apparent that uterine NK (uNK) cells, both uNK1 and uNK2, are significantly enriched in controls, while B cells show the greatest enrichment in cases. As noted in the methods section, these data are corrected for covariates including 10X library batch, sample preparation (whole ME or ME-tissue), nUMI per cell, percent mitochondrial reads and phase. [file 12916_2022_2500_MOESM10_ESM.tiff]

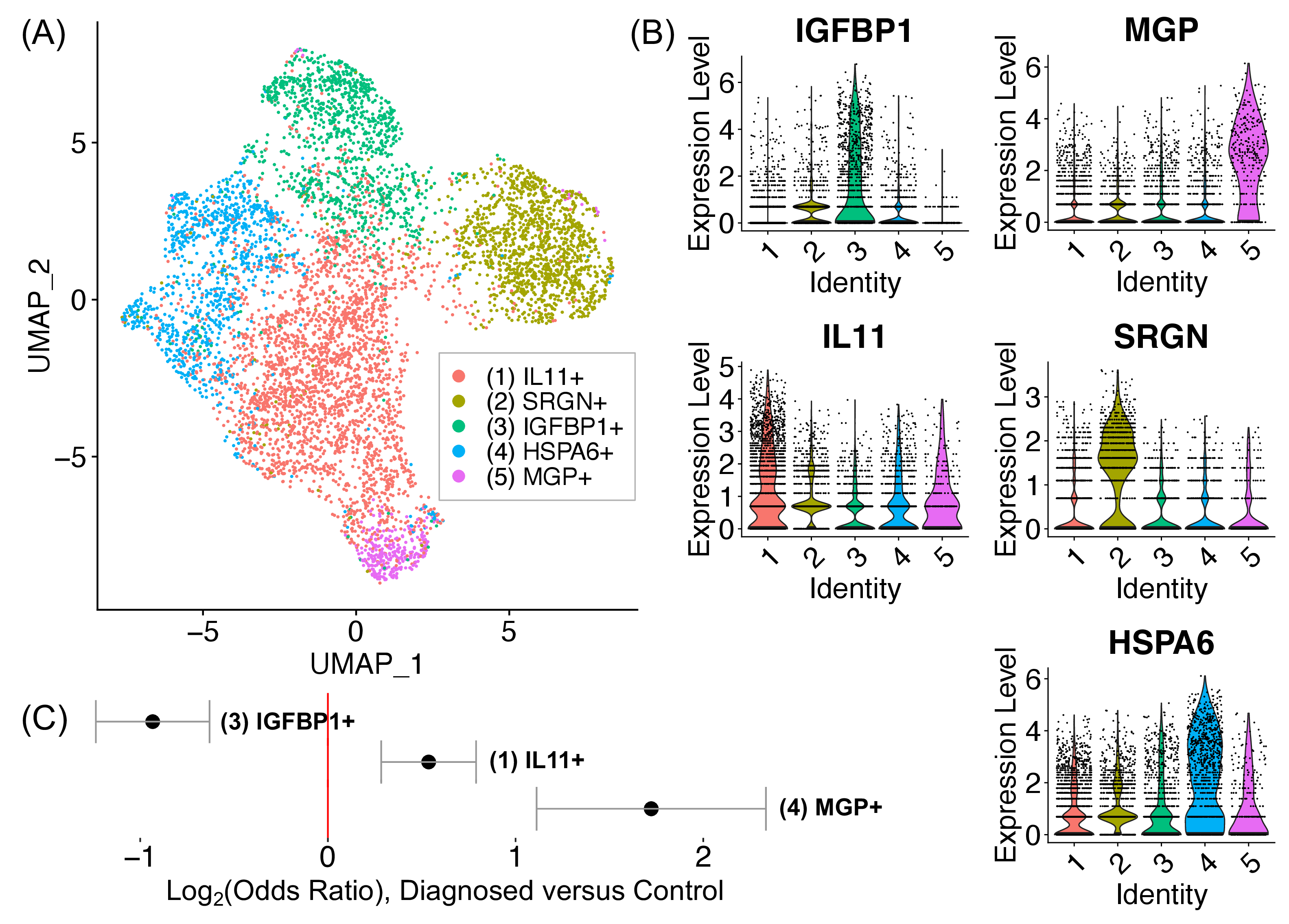

Supplement: Supplementary file 11 — Additional file 11. A reanalysis of stromal cells from endometriosis cases (n=10) and controls (N=9) after removal of one subject on hormones. Not surprisingly, this revised figure shows alterations in the spatial distribution of the UMAP compared to Figure 5 in the main text, but no significant differences in the cell subset distribution comparing cases and controls. [file 12916_2022_2500_MOESM11_ESM.tiff]

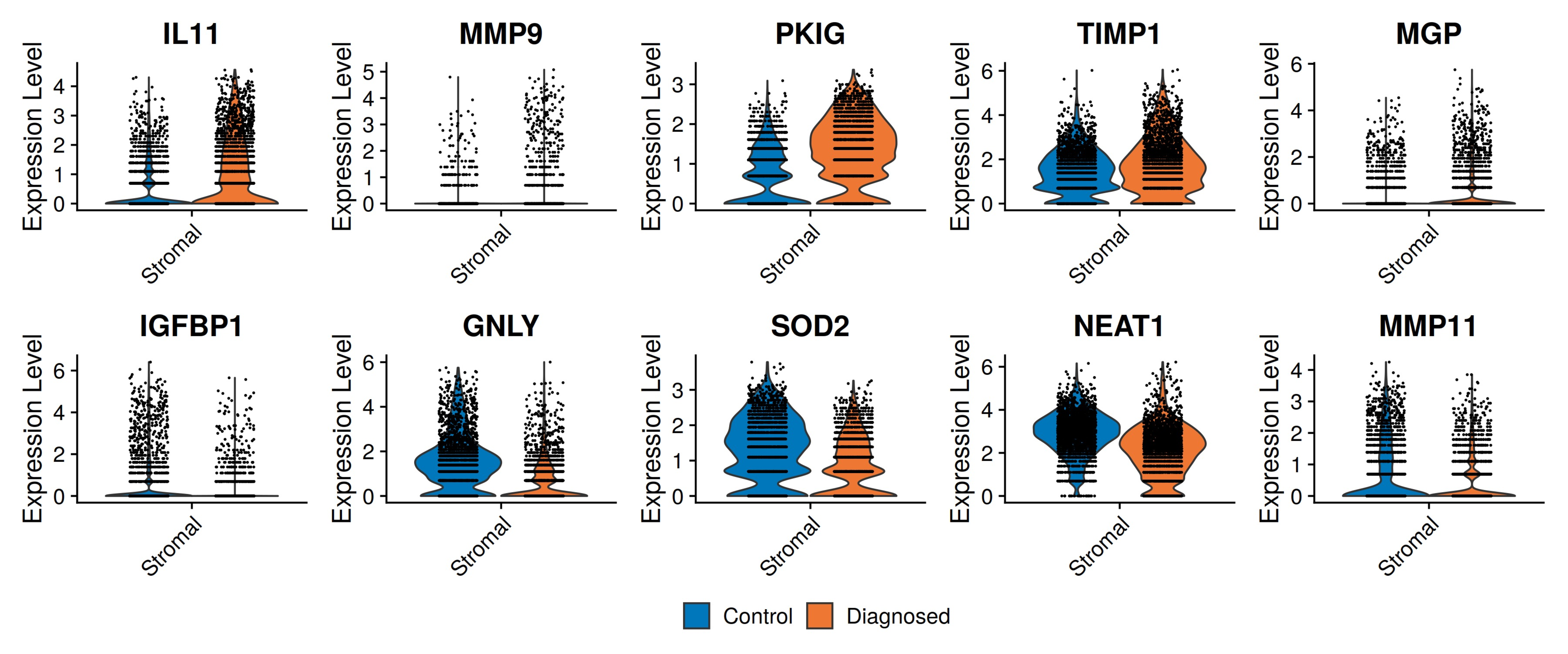

Supplement: Supplementary file 12 — Additional file 12. Stromal cells exhibit distinguishing gene markers differentially regulated in ME from endometriosis cases (n=11) and controls (n=9). Violin plots of the top 10 genes that distinguish endometriosis cases and controls within the total stromal cell population in ME. The data suggest that IL11 and other transcripts may be useful in distinguishing stromal cells isolated from ME obtained from endometriosis case vs control subjects. [file 12916_2022_2500_MOESM12_ESM.tiff]

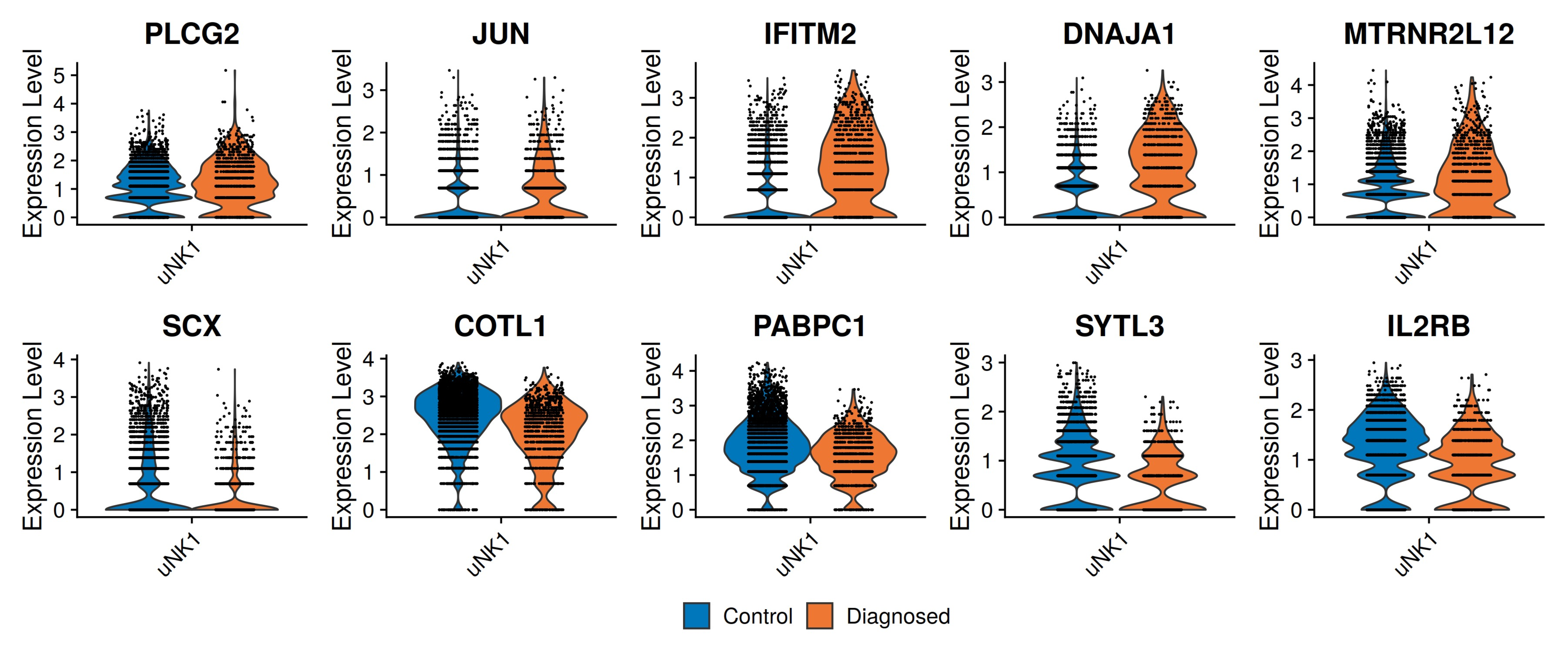

Supplement: Supplementary file 13 — Additional file 13. uNK cells exhibit distinguishing gene markers differentially regulated in ME from endometriosis cases and controls. Violin plots of the top 10 genes that distinguish endometriosis cases (n=11) and controls (n=9) in an analysis of the uNK1 and uNK2 cell subsets as a whole. Note that IFITM2 and DNAJA1 expression are substantially higher in ME obtained from endometriosis cases and may provide a useful diagnostic target based on uNK cells that could be purified from tissues isolated from ME. [file 12916_2022_2500_MOESM13_ESM.tiff]
