# Supplementary material for: Single-cell analysis of menstrual endometrial tissues defines phenotypes associated with endometriosis
Source: BMC Med. 2022 Sep 15;20:315. doi: 10.1186/s12916-022-02500-3 (PMC9476391; doi:10.1186/s12916-022-02500-3)
Supplement: Supplementary file 2 — Additional file 2. Markers for UMAP plots of major cell clusters. [file 12916_2022_2500_MOESM2_ESM.docx]

| **Cell type** | **Associated cluster names** | **Positive marker gene(s) [ref]** |
| --- | --- | --- |
| Uterine natural killer cells | uNK1, uNK2 | *CD45/PTPRC, CD56/NCAM1* [23] |
| Stromal cells | Stromal | *CD73/NT5E, CD90/Thy1, CD105/ENG* [24] |
| CD4+ T cells | CD4T | *CD45/PTPRC, CD3D, CD3E, CD4* [25] |
| Myeloid cells | Myeloid1, Myeloid2, Myeloid3 | *CD45/PTPRC, CD14* [26] |
| Epithelial cells | Epithelial1, Epithelial2, Epithelial3 | CD326/*EPCAM* [27] |
| CD8+ T cells | CD8T1, CD8T2, CD8T3, CD8T4 | CD45/*PTPRC*, CD3D, CD3E, *CD8A* [25] |
| B cells | B | *CD45/PTPRC, CD19, CD20/MS4A1* [28] |
| Plasmacytoid dendritic cells | pDC | *CD45/PTPRC, CD123/IL3RA, CD303/CLEC4C, CD85g/LILRA4* [134] |
| Endothelial-like cells | EC-like | *CD45/PTPRC CD105/ENG, CD31, CD34* [30] |
| Granulocytes | Granulocyte | *GCSAML, CPA3* [9, 10] |
| Unknown Immune cells | Unknown | *CD45/PTPRC* & absence of above gene markers |
