# Supplementary material for: Single-cell analysis of menstrual endometrial tissues defines phenotypes associated with endometriosis
Source: BMC Med. 2022 Sep 15;20:315. doi: 10.1186/s12916-022-02500-3 (PMC9476391; doi:10.1186/s12916-022-02500-3)
Supplement: Supplementary file 5 — Additional file 5. Top 10 genes in groups 2 and 4 stromal cell subclusters from Figs. 5 and 6. [file 12916_2022_2500_MOESM5_ESM.docx]

| **Group 4** | **Group 2** |
| --- | --- |
| *HSPA6* | *SRGN* |
| *NEAT1* | *GNLY* |
| *HSPH1* | *SPP1* |
| *DNAJB1* | *NKG7* |
| *CRYAB* | *CCL4* |
| *HSPA1A* | *GZMA* |
| *HSPB1* | *HLA-DRA* |
| *HSPA1B* | *CD74* |
| *PLCG2* | *CCL3* |
| *HSP90AA1* | *HLA-B* |
