# Supplementary material for: Single-cell analysis of menstrual endometrial tissues defines phenotypes associated with endometriosis
Source: BMC Med. 2022 Sep 15;20:315. doi: 10.1186/s12916-022-02500-3 (PMC9476391; doi:10.1186/s12916-022-02500-3)
Supplement: Supplementary file 6 — Additional file 6. References for genes described in Fig. 6. [file 12916_2022_2500_MOESM6_ESM.docx]

| Gene | **Encodes** | **Estrogen responsive** | **Progesterone responsive** | **Pro-inflammatory/pro-fibrotic** | **Senescence** | **Decidualization**  **Receptivity** |
| --- | --- | --- | --- | --- | --- | --- |
| *IGFBP1* | Insulin-like growth factor binding protein-1 |  | [33, 34] |  |  | [135] |
| *LEFTY2* | Left-Right Determination Factor 2 |  | [36] |  |  | [4,6] |
| *DCN* | Decorin |  | [38] |  |  | [4,8] |
| *MDK* | Midkine |  | [40] |  |  | [40] |
| *LUM* | Lumican | [41] | [41] |  |  | [42, 43] |
| *C1QTNF6* | Complement C1q tumor necrosis factor-related protein 6/CTRP6 |  |  | [12, 13]  Negative CTRP6 attenuates inflammation and fibrosis |  |  |
| *APOD* | Apolipoprotein D | [44] | [45] |  |  | [14,15] |
| *MMP11* | Matrix metalloproteinase 11 |  | [47 ] |  |  | [48] |
| *IGFBP2* | Insulin-like growth factor-binding protein-2 |  | [49] |  |  | [50] |
| *APOE* | Apolipoprotein E |  |  |  |  | [51] |
| *MMP3* | Matrix metalloproteinase-3 | [52] | [53]  P4 Prevents MMP-3 Stimulation | [54, 55] | [56] | [53] |
| *MMP1* | Matrix metalloproteinase-1 | [57] | [58]  Progestins inhibit MMP-1 | [59] | [60, 61] |  |
| *IL11* | Interleukin-11 | [62] | [62]  IL-11 is down-regulated by progesterone. | [63, 64] | [65] | [66]6]  (infertility); blocks trophoblast invasion |
| *SERPINB2* | Serpin Family B Member 2 Plasminogen activator inhibitor-2 | [67] |  | [[68] | [69] | [70] |
| *MMP9* | Matrix metalloproteinase-9 | [71, 72] | [73]  MMP-9 activity is downregulated by P4 | [74] | [75] |  |
| *S100A6* | S100 Calcium Binding Protein A6 | [76] | [77]7P4 inhibits S100A6 expression | [78, 79] | [80]  S100A6 blocks senescence |  |
| *CXCL8* | CXCL8/interleukin-8 | [81] | [82]  P4 withdrawal increases CXCL8 | [83-85] | [86] |  |
| *G0S2* | G0/G1 Switch 2 | [87] |  | [88] |  |  |
| *TMEM158* | Transmembrane Protein 158 |  |  |  | [89] |  |
| *IGFBP5* | Insulin-like growth factor-binding protein-5 | [90, 91] | [33]1]  P4 downregulates | [92] | [93, 94] | [95]  Negative associated with infertility |
| *MGP* | Matrix Gla protein | [68, 96, 97] |  |  | [98] |  |
| *TIMP1* | TIMP Metallopeptidase Inhibitor 1 |  |  |  | [99] | [100]  Neutralizing TIMP1 restores fecundity |
| *TAGLN* | Transgelin | [101] | [102] P4 blocks upregulation of TAGLN by TGFB1/SMAD1 |  | [103]5] |  |
| *COL1A2* | Collagen 1A2 |  | [104] | [105] | [105]7] | [106]  repression of Col1A2 during decidualization |
| *FN1* | Fibronectin 1 | [107] | [108]  P4 withdrawal upregulates fibronectin |  | [105] |  |
| *COL5A2* | Collagen 5A2 |  |  | [84, 109] |  |  |
| *SPARC* | Secreted protein acidic and rich in cysteine |  |  |  | [81,82] |  |
| *COL4A1* | Collagen 4A1 |  |  | [111] | [112] |  |
| *COL1A1* | Collagen 1A1 |  |  |  | [113] | [106]  repression of Col1A1 during decidualization |
| *ACTA2* | a-smooth muscle actin | [136] |  |  | [105, 137] |  |
